# 骨髓涂片胞质轻链免疫荧光结合FISH检测多发性骨髓瘤细胞遗传学异常

**DOI:** 10.3760/cma.j.cn121090-20231204-00291

**Published:** 2024-06

**Authors:** 雨 时, 慧 杨, 睿 郭, 祯 郭, 建勇 李, 雨洁 吴, 海荣 仇

**Affiliations:** 南京医科大学第一附属医院，江苏省人民医院血液科，南京 210029 Department of Hematology, Jiangsu Province Hospital, the First Affiliated Hospital of Nanjing Medical University, Nanjing 210029, China

**Keywords:** 多发性骨髓瘤, 细胞遗传学, 原位杂交，荧光, Multiple myeloma, Cytogenetics, In situ hybridization, fluorescence

## Abstract

**目的:**

分析骨髓涂片胞质轻链免疫荧光结合FISH技术（New-FISH）检测多发性骨髓瘤（MM）细胞遗传学异常的敏感性。

**方法:**

纳入南京医科大学第一附属医院2022年4月至2023年10月收治的42例MM患者，采用组合探针1q21/1p32、p53、IgH、IgH/FGFR3［t（4;14）］、IgH/MAF［t（14;16）］对患者同时进行New-FISH和CD138磁珠分选结合FISH（MACS-FISH）或胞质轻链免疫荧光结合FISH（cIg-FISH）检测，分析其细胞遗传学检测结果。

**结果:**

23例MM患者中，cIg-FISH法、New-FISH法异常检出率分别为95.7％、100.0％（*P*>0.05）。cIg-FISH法、New-FISH法对1q21扩增、1p32缺失、p53缺失、IgH异常的检出率一致，分别为52.2％、8.7％、17.4％、65.2％。进一步对IgH异常的患者进行t（4;14）、t（14;16）检测，t（4;14）阳性率为26.7％，t（14;16）未检出，两种方法检测结果相同。19例MM患者中，MACS-FISH法、New-FISH法异常检出率分别为73.7％、63.2％（*P*>0.05）。MACS-FISH法对1q21扩增、1p32缺失、IgH异常的检出率略高于New-FISH法，但差异均无统计学意义（*P*值均>0.05）。

**结论:**

New-FISH法对于MM患者细胞遗传学异常检出率较高，与MACS-FISH、cIg-FISH一致性较好。

## Abstract (en)

**Objective:**

To analyze the sensitivity of cytoplasmic light-chain immunofluorescence with fluorescence in situ hybridization in bone marrow smears（new FISH）for detecting cytogenetic abnormalities in multiple myeloma（MM）.

**Methods:**

42 MM patients admitted to the First Affiliated Hospital of Nanjing Medical University from April 2022 to October 2023 were enrolled. The patients with MM were detected by new FISH and CD138 immunomagnetic bead sorting technology combined with FISH（MACS-FISH）or cytoplasmic immunoglobulin FISH（cIg-FISH）to analyze cytogenetic detection results using combination probes which included 1q21/1p32, p53, IgH, IgH/FGFR3［t（4;14）］, and IgH/MAF［t（14;16）］.

**Results:**

In 23 patients with MM, the abnormality detection rates of cIg-FISH and new FISH were 95.7％ and 100.0％, respectively（*P*>0.05）. The detection rates of 1q21+, 1p32−, p53 deletion, and IgH abnormalities by cIg-FISH and new FISH were consistent, which were 52.2％, 8.7％, 17.4％, and 65.2％, respectively. The results of the two methods further performed with t（4;14）and t（14;16）in patients with IgH abnormalities were identical. The positive rate of t（4;14）was 26.7％, whereas t（14;16）was not detected. In 19 patients with MM, the abnormality detection rates of MACS-FISH and new FISH were 73.7％ and 63.2％, respectively（*P*>0.05）. The positivity rate of 1q21+, 1p32− and IgH abnormalities detected by MACS-FISH were slightly higher than those detected by new FISH; however, the differences were not statistically significant（all *P* values >0.05）.

**Conclusion:**

The new FISH method has a higher detection rate of cytogenetic abnormalities in patients with MM and has good consistency with MACS-FISH and cIg-FISH.

多发性骨髓瘤（MM）是一种克隆性浆细胞恶性增殖性疾病，骨髓中异常增生的浆细胞分泌单克隆免疫球蛋白或其片段，导致多种器官损伤[1]。蛋白酶体抑制剂、免疫调节药物、单克隆抗体及自体造血干细胞移植等治疗方法的发展显著改善了MM的预后，但疾病的复发、进展仍较为常见，因此评估MM预后并采取最佳治疗策略尤为重要[2]–[3]。遗传学异常是MM的主要预后因素。细胞遗传学核型分析异常检出率约20％，荧光原位杂交（FISH）技术具有较高的敏感性，可提高染色体核型异常检出率。FISH技术以检测MM的1q21扩增、p53缺失、IgH重排、t（4;14）、t（14;16）、t（11;14）等为主[4]–[6]。间期FISH不需要培养染色体中期分裂象，但易受到正常细胞污染，在浆细胞比例低的样本中检出率较低。目前，国内外骨髓瘤工作组均推荐应用CD138磁珠分选结合FISH（MACS-FISH）或胞质轻链免疫荧光结合FISH（cIg-FISH）提高异常检出率[7]–[8]。本研究应用骨髓涂片结合cIg-FISH（New-FISH）对MM常见细胞遗传学异常进行检测，探讨New-FISH对MM细胞遗传学异常的检出率。

## 病例与方法

1. 病例：纳入南京医科大学第一附属医院血液科2022年4月至2023年10月收治的42例MM患者，所有患者的诊断均符合国际骨髓瘤工作组相关诊断标准[9]。其中男21例，女21例，初治34例，治疗后8例，平均年龄67.5岁。临床分型：IgG型24例，IgA型6例，轻链型11例，不分泌型1例。

2. 染色体核型分析：取MM患者的骨髓液培养24 h后，根据标准方案收获骨髓细胞，并通过R显带技术分析核型。依据《国际人类细胞遗传学命名法（ISCN）2020》描述核型异常。每例患者至少分析20个中期分裂细胞，同时，每例标本至少需要2位医师共同鉴定其核型。

3. 单个核细胞提取：取适量淋巴细胞分离液置于离心管中，将患者骨髓液与等量PBS充分混匀后，沿管壁缓慢加于分离液液面上。然后将离心管置于离心机中，720×*g*离心15 min。离心后，管内分为3层：上层为血浆和PBS，下层为红细胞和粒细胞，中层为淋巴细胞分离液，大多数单个核细胞悬浮于上、中层交界处，呈白膜状。吸取单个核细胞置于另一离心管中，加适量PBS，260×*g*离心5 min，弃上清，洗涤细胞3次。将细胞重悬于RPMI 1640培养液中，4 °C保存备用。

4. CD138免疫磁珠分选技术：取2～4 ml患者骨髓液，加入CD138磁珠50 µl/ml，4 °C孵育15 min，离心后去上清，过柱分离浆细胞，洗脱液洗脱分离柱上吸附的浆细胞。收获细胞后，置于4 °C保存备用。

5. 骨髓细胞形态学：骨髓涂片经瑞氏-吉姆萨染色后，对250个有核细胞进行分类，计数各阶段单克隆浆细胞在有核细胞中所占比例。骨髓瘤细胞常成堆分布，大小不一，形态呈多样性；核染色质较细致，有1～2个核仁，可见核畸形、双核、三核或多核浆细胞；胞质丰富，部分骨髓瘤细胞胞质中可见Rusell小体或者空泡。

6. FISH及信号分析：取磁珠分选后的细胞悬液或分离的单个核细胞悬液，使用细胞离心涂片机对两种细胞悬液进行分离制片，41×*g*离心4 min。New-FISH采用未经染色的骨髓涂片，若骨髓涂片已进行瑞氏-吉姆萨染色，需先进行脱色处理。将两种细胞悬液制备完成的玻片及骨髓涂片浸入100％乙醇中固定5 min，然后置于37 °C烘箱中过夜。三种玻片可储存于−20 °C，避免反复冻融。进行FISH检测时，三种玻片在90％甲酰胺溶液中静置5 min，依次经70％、85％、100％乙醇梯度脱水各2 min。晾干后，滴加配制的探针，置于杂交仪中，75 °C变性15 min，42 °C杂交16 h。经50％甲酰胺溶液、SSC缓冲液（2×）、0.1％ NP-40/PBS洗涤。MACS-FISH待玻片晾干后用DAPI进行复染。cIg-FISH及New-FISH进行胞质免疫染色，一抗为山羊抗人κ或λ轻链抗体，二抗为兔抗羊IgG荧光抗体。在荧光显微镜下分析100个浆细胞，计数阳性细胞比例。本实验室5种探针的阈值如下：1q21扩增/1p32缺失探针为20％，p53缺失探针为20％，IgH重排探针为10％，与IgH重排相关的IgH/FGFR3［t（4;14）］、IgH/MAF［t（14;16）］双色双融合检测探针为10％。

7. 统计学处理：采用SPSS 26.0软件进行数据分析，计数资料采用例数（％）描述，计量资料采用*M*（范围）描述，计数资料的比较采用*χ*^2^检验或Fisher精确概率法，*P*<0.05为差异有统计学意义。

## 结果

1. 细胞遗传学及骨髓细胞形态学结果：42例患者中，7例（16.7％）未送检，26例（61.9％）为正常核型，1例（2.4％）为体质性异常，8例（19.0％）为异常复杂核型，其中3例为涉及多条染色体的非克隆性异常核型。骨髓细胞形态学显示，29例（69.0％）患者浆细胞比例≥5％，中位值为14.0％（5.2％～76.4％），浆细胞比例<5％的患者13例（31.0％），中位值为1.6％（0.4％～4.8％）。CD138磁珠分选后的细胞经瑞氏-吉姆萨染色后显示部分细胞被破坏，分选后的细胞包括浆细胞、中性粒细胞等（图1）。

**图1:**
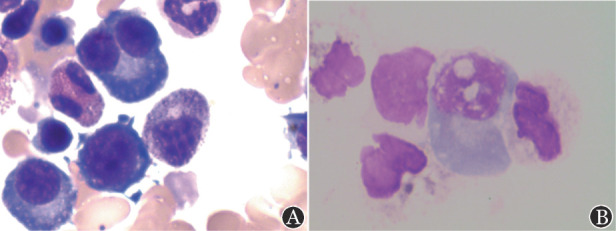
1例患者骨髓涂片细胞形态（A）和骨髓标本经CD138磁珠分选后的细胞形态（B）（瑞氏-吉姆萨染色，×100）

2. New-FISH与cIg-FISH结果比较：同时采用cIg-FISH和New-FISH对23例患者进行1q21扩增/1p32缺失、p53缺失、IgH重排探针检测，结果表明，cIg-FISH组中22例（95.7％）患者有细胞遗传学异常，而New-FISH组中23例（100.0％）患者均检测出异常克隆，两者检出率的差异无统计学意义（*P*>0.05）。两种方法检测1q21扩增的检出率一致（52.2％，12/23），其中1例患者cIg-FISH显示正常而New-FISH检出1q21扩增，另1例患者cIg-FISH显示1q21扩增而New-FISH未检出。cIg-FISH组、New-FISH组1q21扩增阳性细胞比例的中位数分别为57.5％、62.5％。cIg-FISH检测出2例（8.7％）患者具有1p32缺失，异常细胞比例分别为74％、75％；New-FISH检测上述2例阳性患者也均为阳性，异常细胞比例分别为61％、82％。cIg-FISH法、New-FISH同时检出4例（17.4％）p53基因缺失阳性患者，异常细胞比例中位数分别为60％、65％。15例（65.2％）患者存在IgH基因部分缺失、部分扩增及重排，cIg-FISH、New-FISH均检测出异常克隆，异常细胞比例中位数分别为68％、72％。采用两种方法进一步对15例IgH异常的患者进行亚型分析，结果表明4例（26.7％）患者存在IgH/FGFR3［t（4;14）］异常，未检测出IgH/MAF［t（14;16）］异常，cIg-FISH组、New-FISH组t（4;14）异常细胞比例中位数分别为60.5％、83％。New-FISH法检测1q21扩增、1p32缺失、p53缺失、IgH异常、t（4;14）信号清晰，阳性模式典型（图2）。

**图2:**
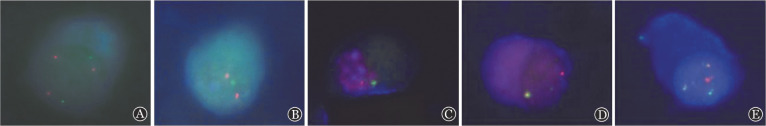
骨髓涂片胞质轻链免疫荧光结合FISH典型荧光信号（×100） **A** 1q21扩增（信号模式：3R2G）；**B** 1p32缺失（信号模式：2R1G）；**C** p53缺失（信号模式：1R1G）；**D** IGH易位（信号模式：1R1G1F）；**E** IgH/FGFR3融合基因（信号模式：1R1G2F） **注** R：红色信号；G：绿色信号；F：黄色信号（融合信号）

3. New-FISH与MACS-FISH结果比较：MACS-FISH法和New-FISH法异常检出率分别为73.7％、63.2％（*P*＝0.485）。两种检测方法均显示异常检出率最高的基因是IgH，MACS-FISH法检测出10例（52.6％）患者具有IgH基因部分缺失、部分扩增及重排，而New-FISH法仅检测出8例（42.1％）阳性患者，异常细胞比例中位数分别为82.5％、49.5％。MACS-FISH法、New-FISH法各检测出7例（36.8％）、6例（31.6％）患者具有1q21扩增，异常细胞比例中位数分别为83％、62％。MACS-FISH法检测出3例（15.8％）患者具有1p32缺失，异常细胞比例中位数为88％；New-FISH法仅检测出2例（10.5％）阳性患者，异常细胞比例分别为66％、67％。两种方法均显示p53缺失阳性患者1例（5.3％）。对IgH异常的患者进行亚型检测，两种方法均检测出3例t（4;14）异常患者。

4. 浆细胞比例与FISH检出率：本研究中骨髓涂片浆细胞≥5％的患者29例，23例同时采用cIg-FISH法和New-FISH法进行检测，有较高的一致性；6例同时采用MACS-FISH和New-FISH，两种方法的结果一致，其中5例阳性，1例阴性。对浆细胞比例较低（<5％）的MM亚组进行分析，13例患者同时采用MACS-FISH和New-FISH，异常检出率分别为69.2％、53.8％（*P*＝0.420）。2例患者MACS-FISH检测出阳性而New-FISH未检出，涉及1q21扩增、1p32缺失及IgH重排，1例患者1q21+、IgH重排阳性细胞比例分别为94％、90％，另1例患者1p32−、IgH重排阳性细胞比例分别为72％、86％，异常细胞比例较高，但浆细胞比例较低，2例患者分别为0.8％及4％。

## 讨论

细胞遗传学是影响MM患者预后的重要因素[10]。染色体核型分析和FISH为美国国立综合癌症网络指南及我国MM遗传学检测专家共识推荐的初诊MM的主要遗传学检测技术。多数MM患者具有复杂异常克隆，但因异常浆细胞有丝分裂指数低，传统的染色体核型分析常显示为正常核型。FISH技术可显著提高染色体核型分析异常的检出率，FISH技术包括间期FISH（D-FISH）、MACS-FISH、cIg-FISH[11]。D-FISH易受正常细胞干扰，呈假阴性，现应用较少。MACS-FISH、cIg-FISH分别通过富集浆细胞及标记浆细胞提高检测的敏感性，是常用的检测方法[12]–[13]。

进行染色体核型分析检测的35例MM患者中，仅发现8例伴异常核型，异常检出率与其他相关报道一致[14]。23例MM患者采用cIg-FISH法检测，异常检出率为95.7％。对19例患者进行MACS-FISH检测，73.7％的患者具有细胞遗传学异常。42例采用New-FISH法检测的MM患者中，83.3％患者检测出异常克隆。三种方法分别通过富集或识别浆细胞减少正常细胞的干扰，异常检出率均较高，但是三种方法各有优缺点。cIg-FISH技术通过提取单个核细胞制片后直接与探针杂交，缩短时间，节约成本，所需的样本量较少。但标本提取过程中会造成部分浆细胞损失，当患者浆细胞比例较低时导致最终判读的浆细胞数量不足。浆细胞含量越低，越需要进行浆细胞富集，本中心前期研究提示，当患者骨髓中浆细胞比例较低时，MACS-FISH检测方法更灵敏。CD138磁珠分选需要特殊的分选磁珠及分选设备，成本较高，费时；由于分选过程中有细胞损失，需要的标本量较多，当浆细胞含量较低时，所需标本量更多。同时，CD138磁珠分选会造成部分浆细胞破裂。本研究采用的新方法无需提取单个核细胞或者磁珠分选，仅需确定患者的轻链类型，选择合适的抗体即可，操作简单；实验室检查中，第一管标本用于骨髓涂片，相较于后抽取的标本，未经稀释，浆细胞比例较高，同时骨髓直接涂片可尽量保持浆细胞形态完整；若MM患者初诊时因诊断不明未留取骨髓标本，需要进行回顾性检测时，可选取留存的骨髓涂片进行New-FISH检测，分析其细胞遗传学异常；若MM患者骨髓浆细胞比例较高，一张骨髓涂片上可进行分区多点杂交。但当骨髓涂片浆细胞比例较低时，结果易呈现假阴性，并且需扩大杂交区域才能计数足够的浆细胞，此时更推荐MACS-FISH检测。

1q21扩增及1p32缺失被认为是MM的独立预后不良因素，其中，1q21扩增是MM患者最常见的细胞遗传学异常[15]–[16]。同时采用cIg-FISH法、New-FISH法的23例患者中，1q21扩增的检出率一致，为52.2％。采用MACS-FISH法、New-FISH法检测的19例患者中，1q21扩增阳性率分别为36.8％、31.6％，略低于相关研究报道[17]。1p32缺失的发生率较低，cIg-FISH法、New-FISH法同时检测出2例MM患者具有1p32缺失，而MACS-FISH法、New-FISH法分别检测出3例、2例。p53基因缺失是高危MM的标志，但其发生率较低[18]。本研究用p53探针对23例患者同时进行cIg-FISH和New-FISH检测，检出率为17.4％；对19例患者同时进行MACS-FISH和New-FISH检测，检出率为5.3％。涉及14q32的易位是MM患者检出率最高的基因异常，与其发生易位的染色体较多，t（11;14）、t（4;14）、t（14;16）较常见，t（4;14）、t（14;16）阳性的MM患者预后不良[19]。本中心在临床检测中发现，当IgH基因出现部分缺失或部分扩增时，也可能与4、11、16号染色体等发生易位。因此，若IgH基因存在上述情况，均进行亚型分析。本研究中，cIg-FISH和New-FISH同时检出65.2％的患者存在IgH基因部分缺失、部分扩增及重排，进一步亚型分析发现，26.7％的患者t（4;14）阳性，未检出t（14;16）。MACS-FISH和New-FISH分别检出10例、8例患者具有上述IgH基因异常，其中，两种方法均检出3例患者具有t（4;14）异常。

本研究首次提出骨髓涂片结合cIg-FISH方法用于MM患者细胞遗传学检测，该方法检出率较高，与现有的MACS-FISH、cIg-FISH方法一致性较好，具有一定的应用前景。但是，在结果分析时，需要准确识别浆细胞并排除杂信号干扰。同时，该方法对涂片质量要求较高，涂片中的细胞需均匀分布。总之，与经典的cIg-FISH及MACS-FISH相比，New-FISH显示出较好的检测能力，是临床中值得推广的检测MM细胞遗传学异常的新方法。
